# Independent processing of increments and decrements in odorant concentration by ON and OFF olfactory receptor neurons

**DOI:** 10.1007/s00359-018-1289-6

**Published:** 2018-09-24

**Authors:** Harald Tichy, Maria Hellwig

**Affiliations:** Department of Neurobiology, Faculty of Life Sciences, University of Vienna, Vienna, Austria

**Keywords:** Insect, Olfaction, Electrophysiology, Antagonism, Concentration

## Abstract

A salient feature of the insect olfactory system is its ability to detect and interpret simultaneously the identity and concentration of an odorant signal along with the temporal stimulus cues that are essential for accurate odorant tracking. The olfactory system of the cockroach utilizes two parallel pathways for encoding of odorant identity and the moment-to-moment succession of odorant concentrations as well as the rate at which concentration changes. This separation originates at the peripheral level of the ORNs (olfactory receptor neurons) which are localized in basiconic and trichoid sensilla. The graded activity of ORNs in the basiconic sensilla provides the variable for the combinatorial representation of odorant identity. The antagonistically responding ON and OFF ORNs in the trichoid sensilla transmit information about concentration increments and decrements with excitatory signals. Each ON and OFF ORN adjusts its gain for odorant concentration and its rate of change to the temporal dynamics of the odorant signal: as the rate of change diminishes, both ORNs improve their sensitivity for the rate of change at the expense of the sensitivity for the instantaneous concentration. This suggests that the ON and OFF ORNs are optimized to detect minute fluctuations or even creeping changes in odorant concentration.

## Introduction

Olfaction is a long-distance sense, vitally important in the natural life of most arthropods. For some insects and marine crustaceans, olfactory cues are much more important than visual or auditory cues in locating food, mates, and shelters. Although odorant sensing seems to be simpler than vision and hearing, orientation in an odorant plume is still insufficiently understood. Unlike wave propagation of visual and acoustic signals, olfactory signals are carried from the source to an animal’s olfactory organ by turbulent plumes. Instantaneously, the plume consists of a series of discrete pulses or patches of varying concentration intermitted by periods of low or zero concentration (Murlis and Jones 1981; Zimmer-Faust et al. 1988, 1995; Moore and Atema 1991). The intermittent structure of turbulent odorant plumes is an important guidance cue to arthropods searching for the odorant source. This is particularly true when combined with other information such as the speed and direction of the flow (reviewed in Webster and Weissburg 2001; Moore and Crimaldi 2004; Koehl 2006; Riffell et al. 2008).

The temporal and spatial distribution of odorant signals in turbulent wind is best described as variations in the frequency, duration, and peak concentration of single odorant pulses (Murlis et al. 1992). This reflects habitat heterogeneity, such as irregularities in the substrate surface or vegetation, or alterations in the flow rate (Finelli 2000). Likewise, in turbulent water currents, the pulse duration and peak concentration show systematic variation across the transverse and longitudinal axes of the plume. At the same time, pulse slope, repetition rate, and the duration of inter-pulse periods or off-times also vary systematically with organism’s position relative to an odorant source (Atema 1985, 1995, 1996; Moore and Crimaldi 2004). With growing distance, odorant plumes tended to expand, decreasing the amplitude of the odorant fluctuations and the steepness of the pulse slopes. These two parameters provide the strongest spatial gradients in turbulent odorant plumes, creating a physicochemical “odorant landscape” (Moore and Atema 1988, 1991; Zimmer-Faust et al. 1995; Finelli et al. 1999).

The relatively large and slow-moving American lobsters use the spatial–temporal distribution of pulse amplitude and slope steepness, or equivalently, the rates of concentration increase to orientate along on odorant plume in turbulent water flow (Atema 1995, 1996; Moore and Atema 1991). Zettler and Atema (1999) were first to demonstrate that the activity of chemoreceptor neurons in the aesthetasc sensilla on the lobster’s lateral filaments varies with the rate of concentration change of the food odorant stimulus. Impulse frequency rose by a factor of 20 with increasing steepness of the onset slope, from 3 imp/s for the gentle slope to 60 imp/s for the steep slope. These experiments provided the initial evidence for the existence of “pulse slope detectors”. A comparison within an ensemble of such “pulse slope detectors”, tuned differently to different rates of concentration increase, would generate different activity patterns depending on the slope steepness, ultimately leading the animal towards the source (Gomez et al. 1999). This concept implies that the rate of concentration change is a quality in its own right, not derived from successive measurements of odorant concentrations. This configuration separates encoding the rate of concentration increase from encoding the sensory qualities supposedly reflected in the activity of these ORNs.

In studies on insect olfaction, variations in the pulse onset slope have not received the same attention as variations in the repetition rates of odorant pulses, the latter commonly tested as trains of transient on–off pulses. Going beyond, pulse coding requires varying the rate of concentration change in a controlled manner and observing the ORN’s activity. This parallels the identification of the “pulse slope detectors” in lobsters (Zettler and Atema 1999). Similar experiments with insects, although technically challenging, would improve our knowledge on the encoding of the rich temporal structure of odorant stimuli. Considering alternatives to the concept of on–off pulses will help generate a broader conceptual framework, including the possibility that the rate of concentration change is encoded in specialized ORNs. Unsolved questions include the range of rates of concentration change that ORNs are able to detect from an almost unlimited number of pulse slopes. The precision with which the response distinguishes different concentration levels could also be addressed. Extracting and encoding biologically relevant temporal information may require highly specialized ORNs or populations of ORNs that combine individual stimulus features of the odorant signal. What is the number of ORNs involved in the detection and encoding of the pulse slopes? What is the relation of ORNs responding and not responding to slow changes in odorant concentration?

To address these issues, we developed a computerized air dilution flow olfactometer capable of delivering both discrete pulsed concentration changes as well as slow and continuous concentration changes at various rates (Burgstaller and Tichy 2011). Our design mimics the rates of concentration increase of odorant pulses within an expanding, less disrupted odorant plume than tested, for example, in studies of pheromone odorant-tracking flights in male moths (Mafra-Neto and Cardé 1995). We used the American cockroach, because it can track a sex pheromone plume in both turbulent and still air, even though the latter process is slower (Willis and Avondet 2005; Willis et al. 2008; Talley 2010). This species has an acute chemical sense; feeding and reproduction are strongly regulated by odorant cues and a wealth of information is available about the olfactory sense (Sakura et al. 2002; Watanabe et al. 2003). The peripheral olfactory system has been extensively studied (Boeckh and Ernst 1987; Boeckh et al. 1990; Seelinger 1990), yielding an almost complete map of olfactory sensilla, innervation patterns, and distributions on the cockroach’s antennae (Sass 1972, 1976, 1978; Altner et al. 1977, 1983; Toh 1977; Schaller 1978; Selzer 1981, 1984; Fujimura et al. 1991). The response spectra of many ORNs to natural food odorants (banana, apple, lemon, orange, bread, meat, and cheese) and to a selected repertoire of chemically pure substances emitted by these odorant sources (alcohols, aldehydes, carboxylic acids, esters, ketones, and terpenes) have been explored in considerable depth using electrophysiological recording techniques. In sum, the olfactory sensilla contain fixed physiological types of ORNs which can easily be identified under the microscope. We tested, for the first time, slowly fluctuating changes in odorant concentration and identified in a structurally distinct sensillum type a pair of ORNs highly sensitive to slow rates of change. They respond to the same change in the concentration of lemon oil odorant, but with the opposite sign (Hinterwirth et al. 2004; Tichy et al. 2005; Burgstaller and Tichy 2011). Slowly increasing concentration raises the impulse frequency in the ON ORN and lowers it in the OFF ORN. Correspondingly, contrary effects are produced by slowly decreasing concentration.

Processing of incremental and decremental lights is a well-studied example of information transfer by parallel ON and OFF pathways. The dual system is formed not at the level of the photoreceptors, which all hyperpolarize to light and have graded potentials, but at the level of the bipolar cells. Bipolar ON cells conserve the signal that they receive from the photoreceptors and bipolar OFF cells invert the photoreceptor signals. Treating luminance increments and decrements independently is a key mechanism in contrast enhancement and edge detection. While the ON and OFF bipolar cells produce transient bursts of action potential emphasizing transient luminance discontinuities in local areas of the retina, the ON and OFF ORNs generate continuous discharges that carry information about both rapid and creeping upward and downward changes of food odorant concentration, respectively.

The present review provides insight into our experimental work on the ON and OFF ORNs of the cockroach’s antenna and gains widely applicable ideas and principles concerning food odorant coding. We address the question of gain control of the ON and OFF ORNs. This control represents a trade-off between sensitivity to the instantaneous odorant concentration and the rate of concentration change. We also describe the effect of the rate of concentration change on the precision of the ON and OFF ORNs to discriminate concentration increments and decrements. We first focus on the classification of the ORNs based on their response characteristics and the observations that morphological sensillum types constantly contain certain physiological classes of ORNs.

## The ON and OFF olfactory receptor neurons (ORNs) are combined in a morphologically defined sensillum type

In some insects, including the cockroach *Periplaneta americana*, the olfactory sensilla are located sufficiently far enough apart on the antennal surface to permit electrophysiological recording from individual, morphologically distinguishable types. This advantage has helped to classify the antennal sensilla using functionally relevant features such as wall structures, presence and location of pores, and numbers of associated ORNs. Schaller (1978) distinguished three types of single-walled sensilla (*swA, swB*, and *swC*) and two types of double-walled sensilla (*dwA* and *dwB*). The *swA* and *swB* sensilla were also known as basiconic sensilla and the *swC* sensillum as trichoid sensillum (Toh 1977). The basiconic *swA* and *swC* sensilla are innervated by two ORNs; the trichoid *swB* sensillum by four.

A large body of evidence amply demonstrates that the basiconic *swB* sensilla and the trichoid *swC* sensilla contain ORNs responsive to citrus fruit odorant. A more detailed analysis revealed that two classes of ORNs located in the basiconic *swB* sensilla produce strong responses to lemon odorant and that nine additional classes produce somewhat weaker responses to the same lemon odorant. The odorant response spectra were determined by presenting the odorants as brief on–off concentration pulses. By contrast, we have tested slow and continuous changes in the concentration of the lemon odorant. The experiments revealed a functional dichotomy of lemon-odorant coding between ORNs in the basiconic *swB* and the trichoid *swC* sensilla. While the ORNs in the *swB* sensilla (~ 27% of the antennal sensilla) did not respond to slow and continuous concentration changes, the ON and OFF ORNs in the *swC* sensilla (~ 6%) did so admirably. The ratio between the sensilla processing information on slow rates of concentration changes and those with other functions is 1:4.5.

**Fig. 1:**
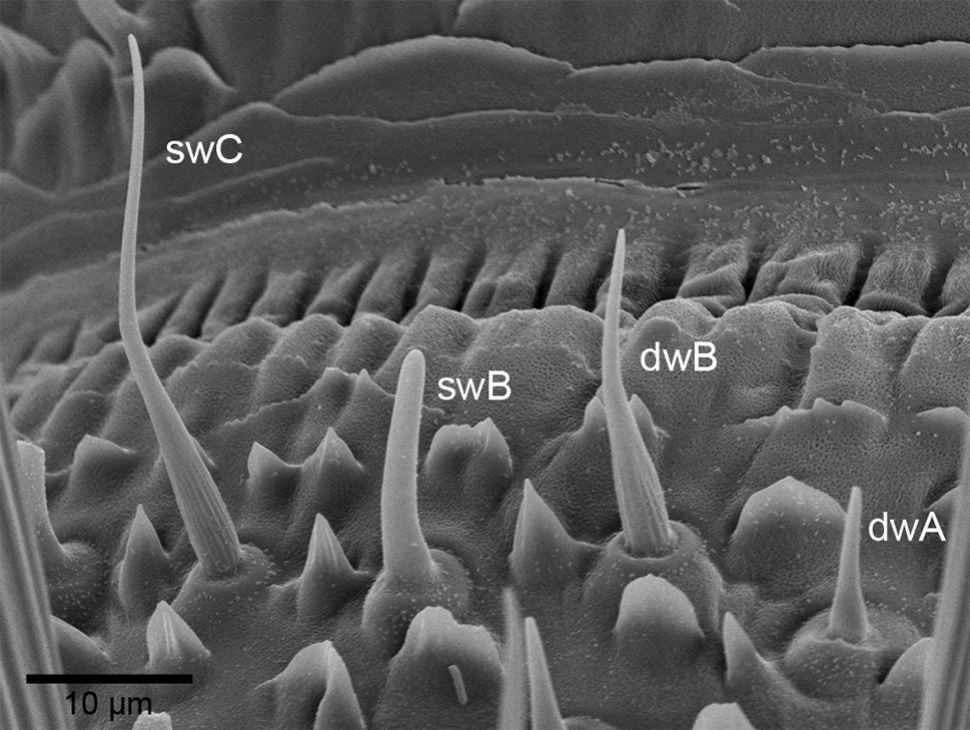
Scanning electron micrograph of different types of olfactory sensilla on the distal margin of a ring-shaped segment in the middle part of the cockroach antenna. The single-walled type C (*swC*) sensillum houses the ON and OFF ORNs. The basal surface of the slightly curved hair is grooved; distally, the surface is smooth and perforated by pores. The shorter single-walled type B (*swB*) sensillum contains four ORNs. The hair is bent and ends bluntly; the wall possesses pores. The double-walled type A and B (*dwA* and *dwB*) sensilla have finely longitudinal grooves into which pore open. Both *dw*-types are associated with four ORNs

**Fig. 2:**
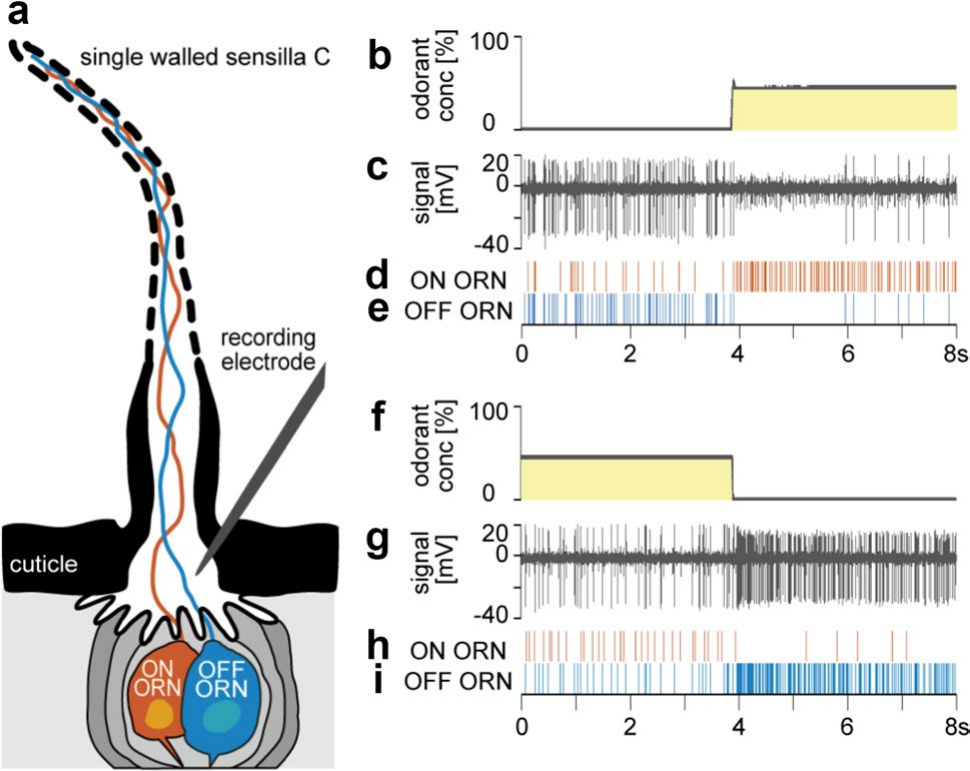
Single-walled type C (*swC*) sensillum containing the ON and OFF ORNs. **a** Diagram of longitudinal section, showing the hair-like cuticular projection, the unbranched dendrites of the two ORNs extending to the tip of the shaft, three auxiliary cells enveloping the ORNs, and the fluid-filled shaft lumen. The recording electrode is gently pushed into the sensillum base. **b** Time course of the concentration of the odor of lemon oil measured by an electronic mass flow meter. Odorant stimulation consisted of a 3-min presentation of clean air, followed by a transient concentration increase at a constant level of 50%, held for 3 min. **c** Simultaneously recorded activity of the ON and OFF ORNs. The OFF ORN typically generates greater impulse amplitudes than the ON ORN. **d, e** Activity of the ON and OFF ORNs displayed in raster plots. **f** Time course of the concentration of the odor of lemon oil measured by an electronic mass flow meter. Odorant stimulation consisted of a 3-min presentation of a 50% concentration level, followed by a transient decrease to clean air. **g** Simultaneously recorded activity of the ON and OFF ORNs. **h**, **i** Activity of the ON and OFF ORNs displayed in raster plots

Figure 1 shows a surface view on the distal margin of an antennal segment, illustrating the location and external structures of the basiconic *swB* and trichoid *swC* sensilla (Schaller 1978; Altner et al. 1983; Hinterwirth et al. 2004; Tichy et al. 2005). Due to the closely adjoining ON and OFF ORNs in the same trichoid *swC* sensillum, the activity of both types could be recorded simultaneously with the same extracellular electrode (Fig. 2a). Differences in the impulse amplitudes enabled unambiguous identification of their responses (Fig. 2c, g). Both ORNs share the same receptive field and, therefore, receive the same concentration change. This proved, beyond all doubts, that the OFF responses are not the result of unfortunate positioning of the antenna in the air stream or turbulence on the virtually laminar flow. One expects that data on the OFF ORNs would be gathered as a by-product of intensively testing the response spectra of food-odorant ORNs. That was not the case. The previous studies applied odorants as series of brief on–off pulses separated by intervals of clean air. This form of stimulation was well suited to produce excitatory responses in the ON ORNs, especially when the durations of the non-stimulus clean-air intervals were long enough to allow adequate conditioning or recovery. Nonetheless, the short concentration pulses, which are the non-stimulus periods for the OFF ORNs, are apparently insufficient to condition the OFF ORNs to the subsequent clean-air interval. An adequate conditioning period prior to the OFF stimulus is necessary to elicit a significant OFF responses. We resolved this problem using an air dilution flow olfactometer, which enables changing the concentration continuously and maintaining it at any level for arbitrary durations.

**Fig. 3:**
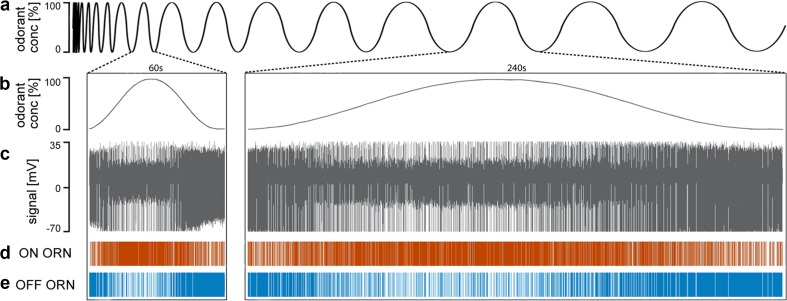
Response antagonism of the ON and OFF ORNs during slowly oscillating changes in the concentration of lemon oil odorant. **a** Time course of the concentration of the odor of lemon oil measured by an electronic mass flow meter. The duration of the oscillation periods was continuously extended from 3 to 360 s. **b** Magnified views of concentration oscillations with periods of 60 s and 240 s. **c** Simultaneously recorded activity of the ON and OFF ORNs. Both types of ORNs discharged continuously during the whole concentration cycle. **d, e** Activity of the ON and OFF ORNs displayed in raster plots

The sharp excitatory response of the OFF ORNs at the end of the odorant pulse (Fig. 2i) might be interpreted as the removal of an inhibitory effect (post-inhibitory rebound) due to the high-concentration odorant pulses. However, the OFF response is not merely a turning off signal. During slow and continuous concentration changes, the OFF ORN discharge also changes slowly and continuously (Fig. 3). Instead of shifting between depolarization and hyperpolarization, the membrane may fluctuate within a range of depolarizations. Intracellular recordings are necessary to determine whether the pauses in the OFF ORN’s discharge at the beginning of the odorant pulse (or the pauses in the ON ORN’s discharge at the termination of odorant pulses; Fig. 2h) are caused by hyperpolarization of the membrane potential. Regarding the transduction process, the molecular driver of the membrane conductance of the ON and OFF ORNs is unknown. Note that both the instantaneous concentration and its rate of change are factors in determining the discharge rate of both ORNs.

Compared to the ORNs in the basiconic *swB*, the ON and OFF ORNs in the trichoid *swC* sensilla are continuously active as long as the concentration of the air stream remains constant. Even in still air, both ORNs have an unceasing resting discharge (Burgstaller and Tichy 2012). No information is available on the mechanism by which this resting or spontaneous discharge is maintained during exposure to a constant concentration, including zero concentration. It is improbable that “spontaneous” excitation is suited for explaining the incessant activity. Its origin may be intrinsic to the ORN and modulated by the receptor potential due to the fluctuating odorant concentration. Experiments on the warm receptor neurons of the tarsal organ of the wandering spider *Cupiennius salei* provided conclusive evidence that their continuous activity persists when the sensory input is blocked with Epoxy glue (Gingl and Tichy 2006). The toxic cyanoacrylates in the glue are irreversible and unspecific membrane channel blockers, apparently selectively poisoning the receptive terminals and preventing conductance changes necessary for the receptor potential. The mechanisms responsible for the action potentials were not eliminated by locally applying this glue. Electrical and temperature stimulation of the warm receptor neurons with blocked and unblocked receptive region indicates that the continuous discharge is equally modulated by the receptor potential due to rapidly rising ambient temperature as it is by current application.

Furthermore, the warm receptor neurons are highly sensitive to low-amplitude temperature changes (Ehn and Tichy 1996). This ability is conceivable only if their discharge was perfectly regular, then any departure from the continuous activity would signal a temperature change. Detecting slight temperature changes is improved by adding the low-frequency receptor potential (caused by the temperature change) to a higher frequency carrier signal, a process known as frequency modulation. In the warm receptor neuron, the unmodulated carrier frequency corresponds with a pacemaking discharge. When the modulating receptor potential is applied, the discharge frequency will swing above and below the carrier frequency according to the amplitude of the modulating receptor potential. Any deviation from this discharge rhythm signals a change. Threshold is not defined as the discharge itself, but as a change in its prevailing rate. One future task would be to determine whether the ON and OFF ORNs are their own pacemakers, as postulated for spider warm receptor neurons, whose activity persists without external drive.

It is worth noting that ephaptic coupling or non-synaptic inhibition, described in *Drosophila* for ORNs and CO_2_ receptor neurons located in the same sensilla (Su et al. 2012), does not correspond with the ON and OFF responses. The ORNs responsible for ephaptic coupling share no direct connections, as generally assumed for ORNs arranged as pairs in the various olfactory sensilla. Nevertheless, these ORNs can stop each other reciprocally from discharging. In ephaptic coupling, the maintained activity of one ORN to the prolonged presentation of a background odorant was suddenly inhibited by a brief, superimposed pulse of a different odorant. The latter elicited a strong excitatory response in the second ORN. Two points should be borne in mind. First, ephaptic coupling was found in a two-odorant paradigm, but the antagonistic ON and OFF responses occur in a one-odorant paradigm. Second, the ON and OFF responses can be elicited by both transient and slow concentration changes. The question remains whether ephaptic coupling is limited to transient concentration changes. Future intracellular recordings should clarify whether the ORNs are truly inhibited by ephaptic coupling, i.e., the membrane is hyperpolarized by the odorant pulse. An alternative explanation is that the discharge pause in one ORN is caused by the removal of ions from the receptor lymph due to the increased conductance and depolarization of the other ORN. This alternative is corroborated by the fact that ephaptic coupling is associated solely with interrupted discharges but not with excitation.

Rapid concentration changes are clearly useful stimuli. By rapidly passing the excitation threshold, the discharge rate far outweighs the neural noise. In contrast, slowly fluctuating or creeping concentration changes make it difficult to decide whether the neural activity results from noise alone or from a response plus noise. The next section analyses the effects of slow concentration changes on the discharge rates of the ON and OFF ORNs.

## Double dependence on instantaneous odorant concentration and its rate of change

The segregation of olfactory input by ON and OFF ORNs provides a means for transmitting information about concentration increment and decrement with an excitatory process to the brain. It must be determined how much information is available from processing the momentary state of excitation of each type of ORN. Temporal fluctuations in odorant concentration provide an important directional cue that insects detect and use during source location in a turbulent odorant plume. To correctly assess the instantaneous distribution of the odorant signal, the intervals between the pulses are as meaningful as the pulses themselves. Indeed, ON and OFF durations are key events in a turbulent odorant plume and directly signaled by the ON and OFF ORNs.

There are also other, even more complex aspects to odorant signal detection, such as the rate with which concentration changes. Any analysis of ORN responses typically first examines average values to describe pulse concentration and then provides the details of the odorant signal, including the time course. Our approach, however, was to initially focus on moment-to-moment change in odorant concentration and then delve into different methods to extract information, including averaging. No matter how the data are finally presented; time is the common abscissa. When plotting the ORN’s impulse frequency and the changing odorant concentration as a function of time, the rate of concentration change is of course implied in the time course of odorant concentration. Importantly, the values measured for the instantaneous concentration apply directly to the stimulating air stream and less directly to the sensillum or to the receptive site of the ORNs. Assigning instantaneous concentration values to the receptive sites is possible only at low rates of concentration change. This enables correlating impulse frequency with instantaneous concentration values at the receptive sites during concentration changes and also with accurate values for the rate of concentration change.

**Fig. 4:**
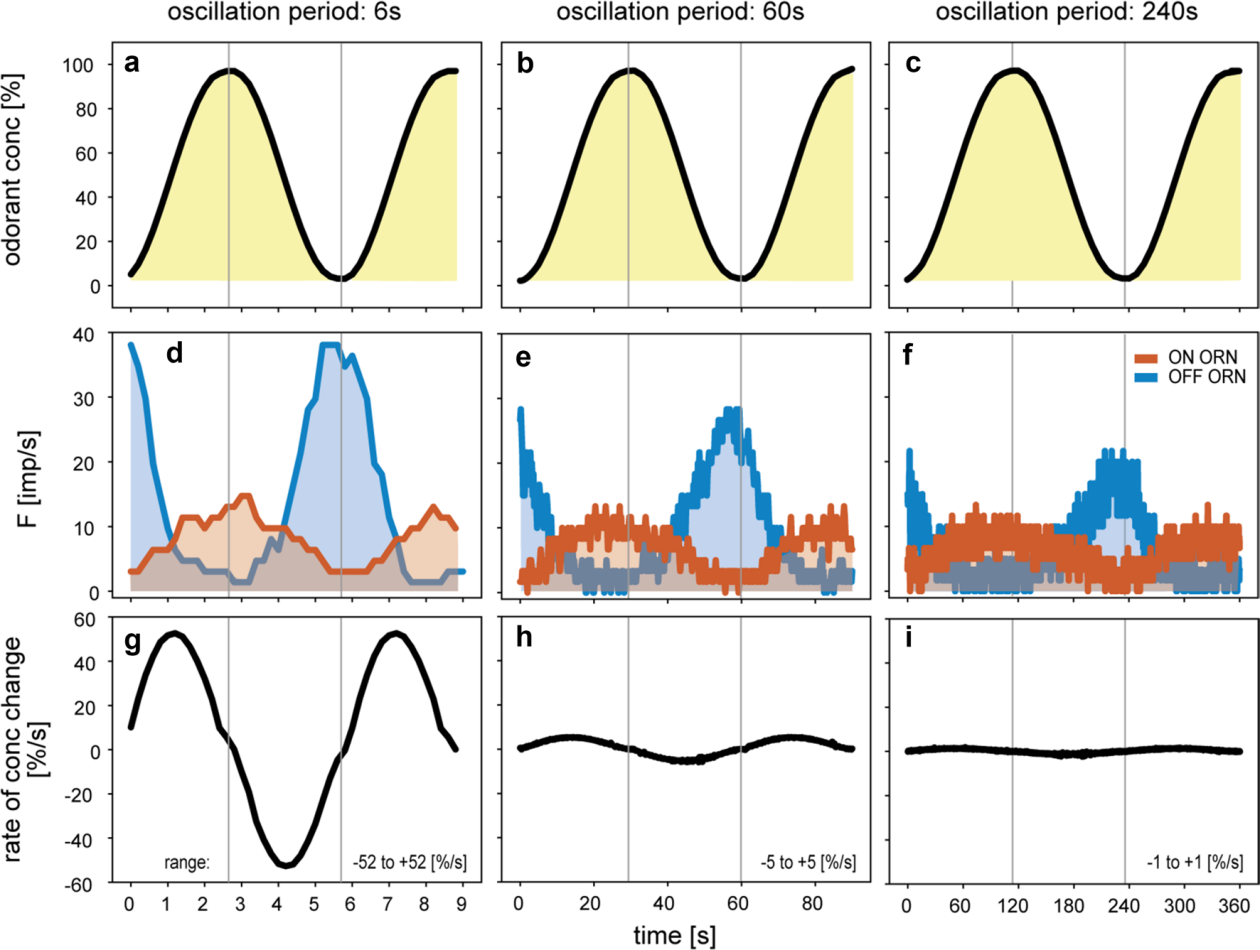
Responses of a pair of ON and OFF ORNs recorded simultaneously from the same sensillum during slowly oscillating concentration changes with periods of 6 s, 60 s, and 240 s. **a**–**c** Time courses of odorant concentration. **d**–**f** Time courses of impulse frequencies of the ON ORN (orange) and OFF ORN (blue). **g**–**i** Time courses of the rate of concentration change. Concentration range was slightly smaller than 100%; the rate of concentration change was between ± 52%/s during the 6-s oscillation period and ± 1%/s during the 240-s oscillation period. Vertical lines indicate phase differences between oscillations in odorant concentration, oscillations in impulse frequencies, and oscillations in the rate of concentration change. Impulse frequencies of both ORNs are in advance of instantaneous odorant concentration and behind its rate of change. Impulse frequency (*F*) determined for 1 s periods. Adapted from Burgstaller and Tichy (2012)

To vary the instantaneous concentration and its rate of change independently of each other, we produced oscillating concentration changes and altered the oscillation period between 3 and 360 s. The rate of concentration change ranged from 2%/s during 360-s oscillation periods to 105%/s during 3-s oscillation periods (Fig. 3). The ON ORNs always showed one clear frequency maximum per concentration maximum, and the OFF ORNs, one per concentration minimum. This relationship suggests that the discharge of both ORNs carries information on fluctuating concentration changes. Figure 4 illustrates some of the parameters that govern the responses. The diagrams in the top row (Fig. 4a–c) show the time course of concentration oscillations with periods of 6 s, 60 s, and 240 s; in the middle row (Fig. 4d–f) the oscillations in impulse frequency of the ON and OFF ORN; in the bottom row the oscillations in the rate of concentration change (Fig. 4g–i). With increasing duration of the oscillation period and decreasing rate of concentration change, impulse frequencies of both ORN types decreased continuously, even though the concentration range was the same. The oscillating frequency of the ON ORN seems to match the oscillations in odorant concentration, and the OFF ORN’s frequency oscillations to mirror the concentration oscillations. However, there are two important differences. First, the activity of the OFF ORN spans a larger frequency range than the ON ORNs, which indicates that the former responds with higher frequencies to concentration decrements than the latter to equally strong concentration increments. Second, impulse frequencies of both types of ORNs lead to odorant concentration, whereas, during brief oscillation periods (3–18 s), the frequency oscillations of both ORNs are in step with the concentration oscillations (Fig. 4d); during periods exceeding 30 s, the frequency oscillations precede the concentration oscillations (Fig. 4e, f). Thus, both ON and OFF ORNs depend not only on concentration. The rate of concentration change is the obvious choice of an additional stimulus parameter (Fig. 4g–i). As the first-order derivative of concentration, this rate of change precedes the instantaneous concentration, and no other parameter was varied in the experiments. During brief oscillation periods, the frequency oscillations of both ORNs are in phase with the oscillating rate of concentration change, but, during periods exceeding 30 s, they lagged behind. The duration of the lag increases with the duration of the oscillation period. Thus, the oscillations in impulse frequency are between those of instantaneous odorant concentration and its rate of change. Both parameters of the odorant stimulus govern the ORN responses.

**Fig. 5:**
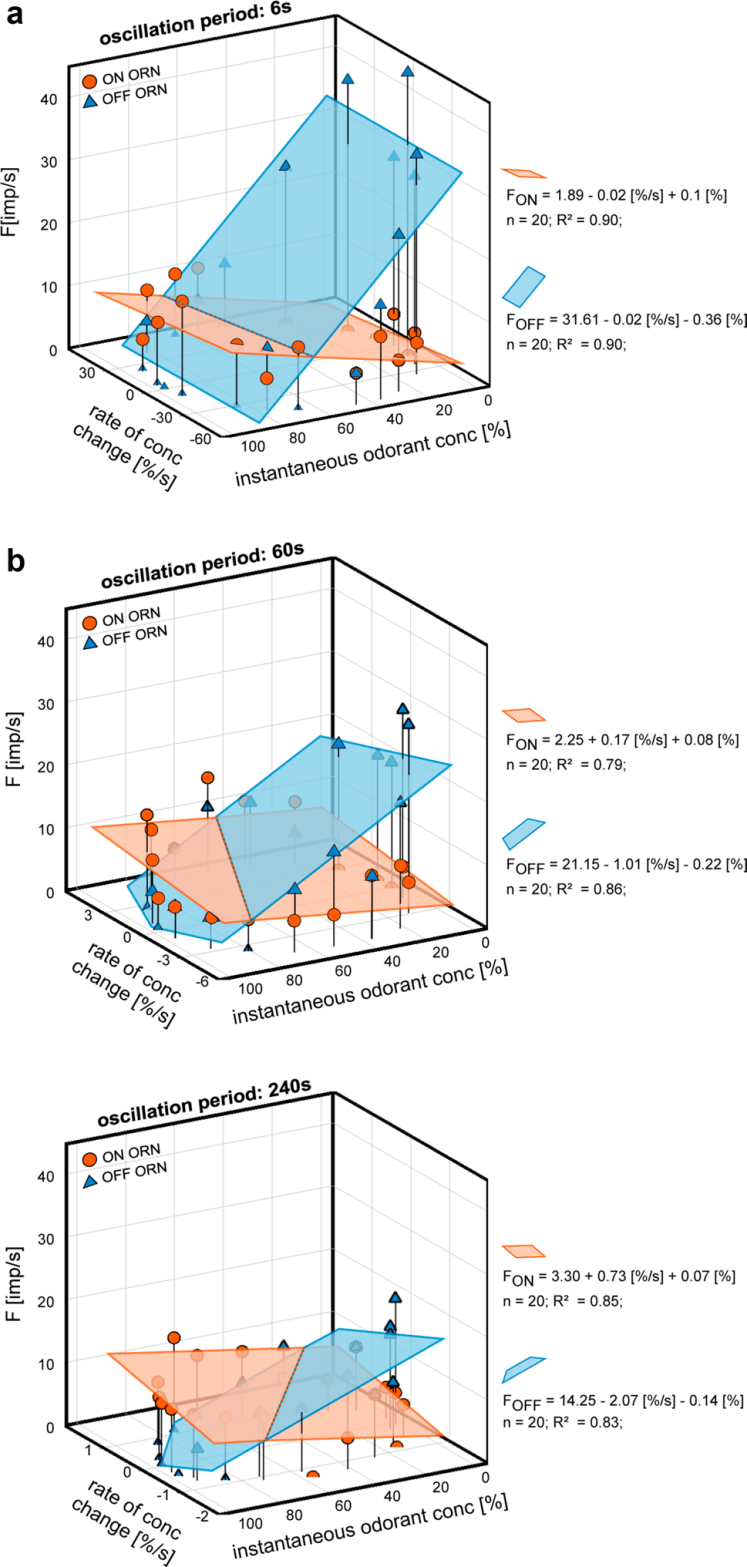
Responses of a pair of ON ORN (orange) and OFF ORN (blue) recorded simultaneously from the same sensillum during slowly oscillating concentration changes with periods of 6 s, 60 s, and 240 s. Impulse frequencies plotted as functions of the instantaneous odorant concentration and its rate of change. Regression planes [*F* = *y*_0_ + *a*(d*C*/d*t*) + *bC*; where *F* is the impulse frequency and *y*_0_ is the height of the regression plane] were computed to estimate the gain for instantaneous odorant concentration (*b*-slope) and the gain for the rate of concentration change (*a*-slope). Impulse frequency (*F*) of both ORNs increases with both the instantaneous concentration (*C*) and its rate of change (d*C*/d*t*), with due consideration of sign. *R*^2^, coefficient of determination; the number of points per plot, *n*, was 20. Adapted from Burgstaller and Tichy (2012)

The 3D scatter plots in Fig. 5 enable a more precise view on the simultaneous dependence of both types of ORNs on the instantaneous concentration and its rate of change. Comparing the discharge rates of individual ORNs during a single oscillation period shows that impulse frequency is not the same when ascending or descending through the same concentration value. The impulse frequency displays such a pronounced hysteresis that the closed figures approached circles. This type of response is not new. It has previously reported in thermoreceptors during slowly increasing and decreasing ambient temperature or infrared radiation (Gingl and Tichy 2001; Fischer and Tichy 2002; Gingl et al. 2005; Tichy et al. 2008; Zopf et al. 2014; Tichy and Zopf 2015) and in hygroreceptors during slowly increasing and decreasing ambient humidity or air pressure (Tichy 2003; Tichy and Kallina 2010, 2013).

An important characteristic of the ORNs is the differential sensitivity or the gain of responses, usually defined as the ratio of the output impulse frequency to the input concentration of the odorant stimulus. This value is indicated by the slopes of the best-fit regression planes (Fig. 5). In both ORNs, the steepness of the regression slopes depends on the duration of the oscillation period. The positive ON ORN slopes reveal that impulse frequency is high when odorant concentration is high and even higher when that concentration is also rising. Conversely, the negative OFF ORN slopes indicate a high impulse frequency at low odorant concentration and an even higher one when that odorant concentration is also falling. The effect of the instantaneous odorant concentration on all ORN responses is reinforced by the rate of concentration change. The gain values of the OFF ORN tend to be higher than those of the ON ORN. Concentration decrements are more important than concentration increments in the search performance for odorant sources.

The ON and OFF ORNs are not symmetrically responding to the same changes in odorant concentrations. Since both types respond differently to the instantaneous concentration and its rate of change, proper central processing of parameters may yield unambiguous information concerning either. Note that the periods of oscillating concentration changes ranged from 3 to 360 s. Periods shorter than 3 s were omitted, because it was difficult to adjust the valves, so that the odorant-loaded air flows smoothly rather than abruptly over the antenna. The shortest possible oscillation periods that can be detected and differentiated by the ON and OFF ORNs remain unknown. Nonetheless, the resolution of brief on–off pulses was studied in different insects and marine crustaceans. The rates at which odorant pulses still elicit distinguishable bursts of action potentials are species specific and ranged from 5 to 50 Hz (Weissburg 2000; Szyszka et al. 2014). In the American cockroach, ORNs followed 25-ms pulses of 1-hexanol up to rates of 40 Hz and 50-ms pulses of coconut oil up to 20 Hz (Lemon and Getz 1997). The ORNs apparently update the representation of such stimuli every few tenths of seconds, a period comparable with the retinal ganglion cells (Meister and Berry 1999). Electroantennogram (EAG) recordings, which represent the summed potential of all electrical activity of an intact but excised antenna, revealed a maximum resolution of 3-ms pulses of 2-heptanone at 50 Hz in the hissing (orange spotted) cockroach and 125 Hz in the honey bee and locust (Szyszka et al. 2014). These studies showed that a sequence of equal-concentration on–off pulses elicit a corresponding sequence of burst-like responses, but impulse frequency within the bursts decreased with progressive pulse repetition rather than remaining at a stable level (Lemon and Getz 1997). The EAG amplitude also declined with increasing repetition rate (Szyszka et al. 2014). This decrease in response magnitude may reflect adaptation or fatigue, indicating that the ability to resolve a rapid sequence of odorant pulses does not necessarily correspond with the precise detection of pulse concentration. The role of these ORNs could be to signal quick successions of transient concentration changes rather than to provide precise information on the concentration of the transient change. The instantaneous detection of odorant fluctuations may only be possible at slower repetition rates.

Most physiological experiments failed to quantify the rate of concentration increase of the odorant stimulus. More recently, however, a photoionization detector (PID) was routinely employed to monitor and measure the gas-phase concentration of the square-shaped odorant pulses. In *Drosophila*, the time course of on–off concentration pulses deviated from a square pulse (Martelli et al. 2013). In particular, the onset slopes of the PID signals differed depending on the odorant. Accordingly, odorant stimuli were divided into “fast odorants” with steep onset slopes (methyl butyrate, 1-pentanol, and propylene acetate), and “slow odorants” with longer rising times (1-octen-3-ol, diethyl succinate). The normalized PID signal shows that the pulse shape of “fast odorants” is unaffected by odorant concentration, but, in the “slow odorants”, this shape does change with odorant concentration. The response of individual ORNs to the two groups of odorants differs greatly in magnitude and time course. One explanation is that impulse frequency reflects not only odorant identity and concentration but also odorant pulse shape or, more precisely, the pulse onset slope. The question of whether these ORN detects and encodes the temporal concentration profile of the odorant stimulus could be answered in the future by delivering “slow odorants” with fast rise times and “fast odorants” with slow rise times.

Simultaneous recordings from pairs of directly connected ORNs and projection neurons (PNs) in the antennal lobe of *Drosophila* revealed that some PNs contain not only information on the incoming rate of discharge of the ORN but also on the rate at which the ORN’s discharge rate is changing (Kim et al. 2011, 2015). The finding that PNs signals accelerating ORN activity suggests that the ORNs can detect and encode the rate of change of the stimulus concentration. During transient on–off concentration changes, the values of concentration acceleration were great. In contrast, during slow and continuous concentration changes, the acceleration was slow. The conclusion is that there is some low, as yet untested rate of concentration change at which the ORN discharge rate is not transformed into acceleration by PNs. The threshold of transformation can be determined by gradually varying the rate of concentration change from slow to fast.

*Drosophila* provides one more example for the rate of concentration change being a factor in determining PN activity (Bhandawat et al. 2007). The discharge rate elicited by brief on–off pulses increases more rapidly in PNs than in ORNs, decaying earlier in PNs. The frequency peaks of the ORNs and PNs are not in phase; the latter precede the former. Those authors interpreted this phase change as an indication that PNs activity represents the rate at which ORN discharge is changing. Unfortunately, they did not measure the time course of the concentration pulses and did not vary the rate of concentration change. Further research is required to determine the range of rates of concentration change of odorant pulses which these ORNs can encode.

We have shown that the cockroach’s ORNs directly combine two independent stimulus parameters: the instantaneous concentration and its rate of change. The manner of combination is addressed in the next section.

## The rate of concentration change acts as gain control signal

**Fig. 6:**
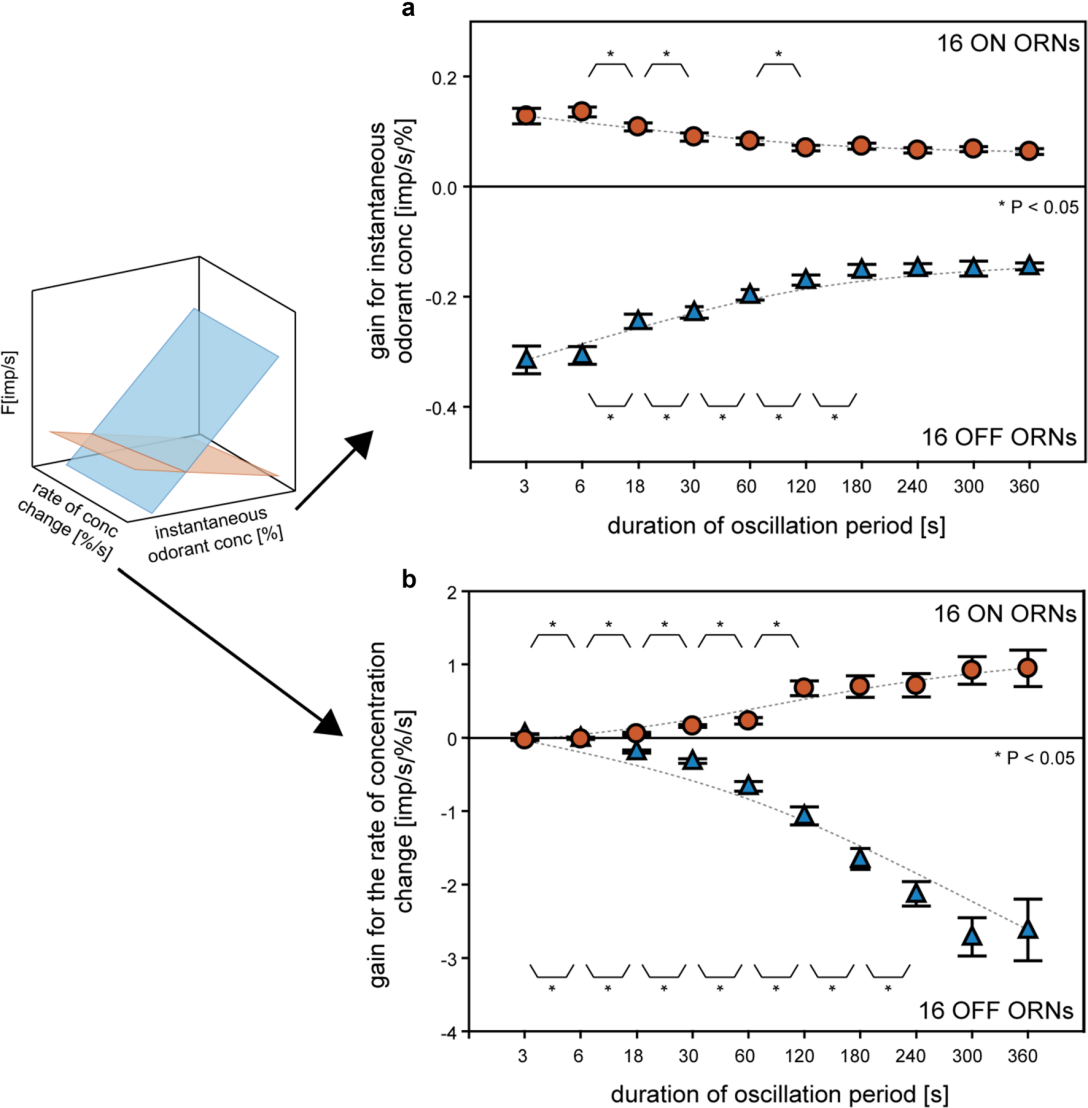
Effect of the duration of the oscillation period of the gain values of ON ORNs (orange) and OFF ORNs (blue) for the instantaneous odorant concentration and its rate of change. **a** In both types of ORNs, the gain for the instantaneous odorant concentration decreases, sign ignored, with increasing duration of the oscillation period. **b** Conversely, the gain of both ORNs for the rate of concentration change increases when the period length increases, with due consideration of the sign. The negative gain values are due to the downward direction of the concentration change, resulting in an increase in impulse frequency of the OFF ORN. Mean values and standard deviation attained from 16 ON and 16 OFF ORNs are indicated. The number of measurements, *n*, used to determine gain is 16 for the oscillation periods in the range between 3 and 120 s, *n* = 14 for the 180-s period, *n* = 12 for the 240-s period, *n* = 10 for the 300-s period, and *n* = 8 for the 360-s period. Differences in gain values between adjoining oscillation periods were tested for statistical significance by the paired Student’s *t* test, *P < 0.05. For periods with *n* < 14, significance was not tested. Adapted from Burgstaller and Tichy (2012)

**Fig. 7:**
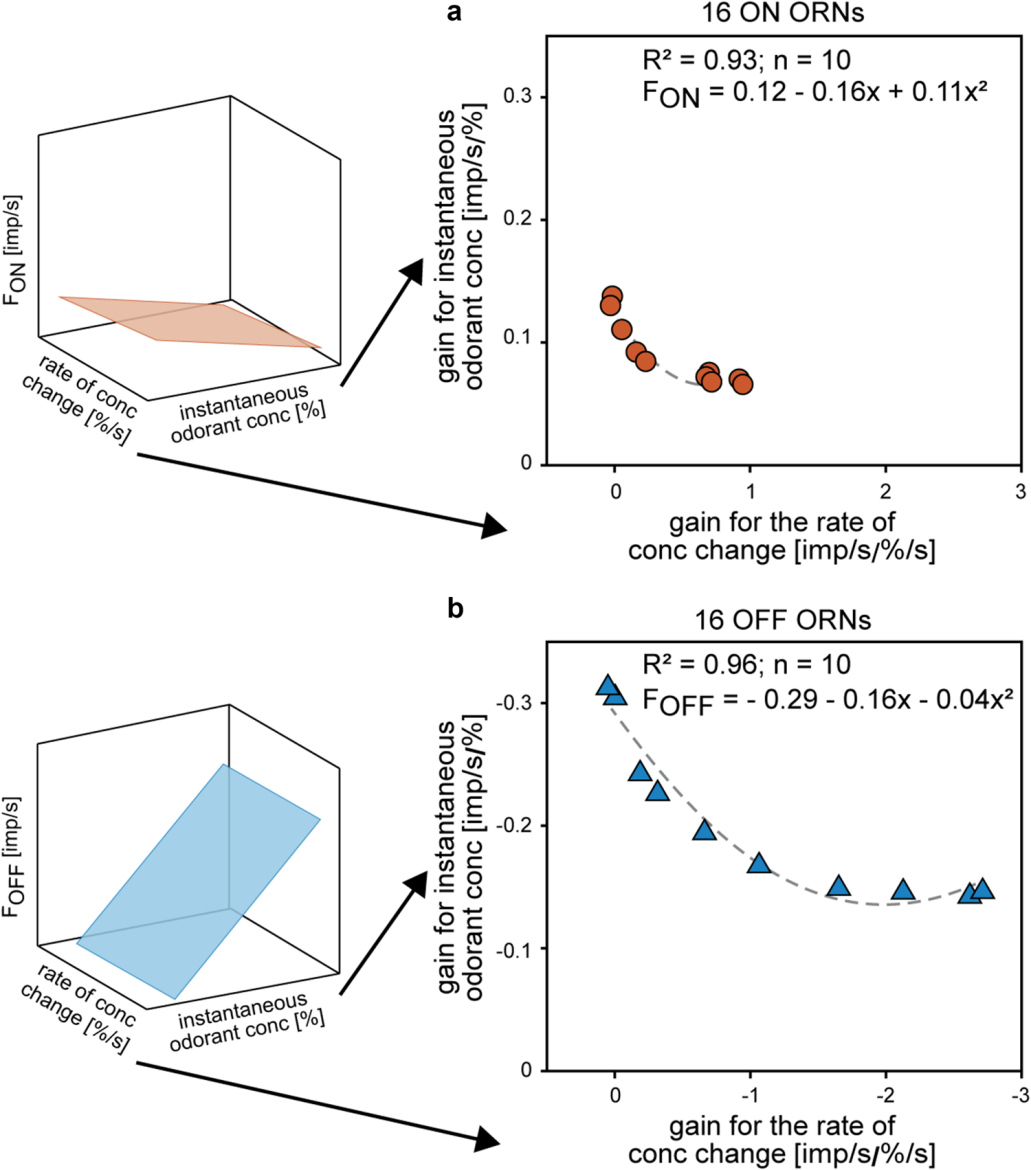
Correlation between the response gain for instantaneous concentration and the response gain for the rate of concentration change of the ON ORN (orange) and OFF ORN (blue) determined for ten oscillation periods in the range of 6–360 s. In both types of ORNs, the decrease in the gain for instantaneous concentration is related to the increase in the gain for the rate of concentration change. The negative gain values are due to the downward direction of the concentration change, resulting in an increase in impulse frequency of the OFF ORN. Mean gain values of ten ORNs are indicated and used to estimate the relations by exponential functions. Adapted from Burgstaller and Tichy (2012)

A striking property of the ON and OFF ORNs is their ability to operate over a wide range of rates of upward and downward concentration changes, respectively. This flexibility is enabled by adaptation mechanisms that dynamically control the differential sensitivity or gain of responses (Burgstaller and Tichy 2012). Gain control involves a trade-off between sensitivity to instantaneous odorant concentration and sensitivity to the rate of change (Fig. 6). The gain of both ORN types for the instantaneous concentration declines, sign ignored, when the oscillation period is extended from 6 to 60 s. Further extending the period yields a constantly low gain value (Fig. 6a). Conversely, the gain values for the rate of concentration change increases when the period is increased, with due consideration of the sign (Fig. 6b). During slow oscillations with long periods, both ORNs improve the gain for the rate of change at the expense of the gain for instantaneous concentration. This enables the ON and OFF ORNs not only to keep up with both rapid and slow fluctuations in odorant concentration, but also to balance—from instant to instant—their sensitivity according to the rate at which concentration changes (Fig. 7). During rapid oscillations, gain control prevents the ORNs from reaching saturation and decreasing sensitive for concentration increments and decrements. During slow oscillations, cockroaches need to determine whether the discharge rate is changing at all. Because of the high gain for low rates of change, the ON and OFF ORNs are best suited for detecting and processing slow concentration changes, even if they maintain for several minutes without changing directions. Gain control provides high precision for slow rates when this is vital, without narrowing the detectable and useable concentration range during orientation and without expansion of the response scale.

This type of gain control is unique to cockroach’s ORNs today. Future studies should investigate other insects in this regard. Nevertheless, gain control has been described in different insects as a mechanism by which higher order olfactory neurons in the antennal lobe combine and process information about on–off concentration pulses. In the migratory locust, for example, the total olfactory input to the antennal lobe increases with odorant concentration, but the total output of the antennal lobe, integrated across the population of projection neurons, does not exceed a 1000-fold increase in odorant concentration (Stopfer et al. 2003). Gain control adjusts the input–output functions of the locusts’ antennal lobe, preventing PN saturation. Another gain control mechanism has been described for the olfactory circuit in the antennal lobe of *Drosophila*. Presynaptic inhibition by local neurons suppresses ORN discharge rates at high concentrations (Olsen and Wilson 2008; Oizumi et al. 2012). This process was interpreted twofold: in relation to a concentration-independent representation of odorant identity, and prevention of response saturation at higher concentrations (Asahina et al. 2009; Olsen et al. 2010).

Now, it has been demonstrated that the ON and OFF ORNs regulate their sensitivity according to the rate of concentration increments and decrements, respectively. The next section describes how the rate of change governs the ability of both ORNs to discriminate instantaneous concentration levels.

## The rate of concentration changes determines the resolving power

**Fig. 8:**
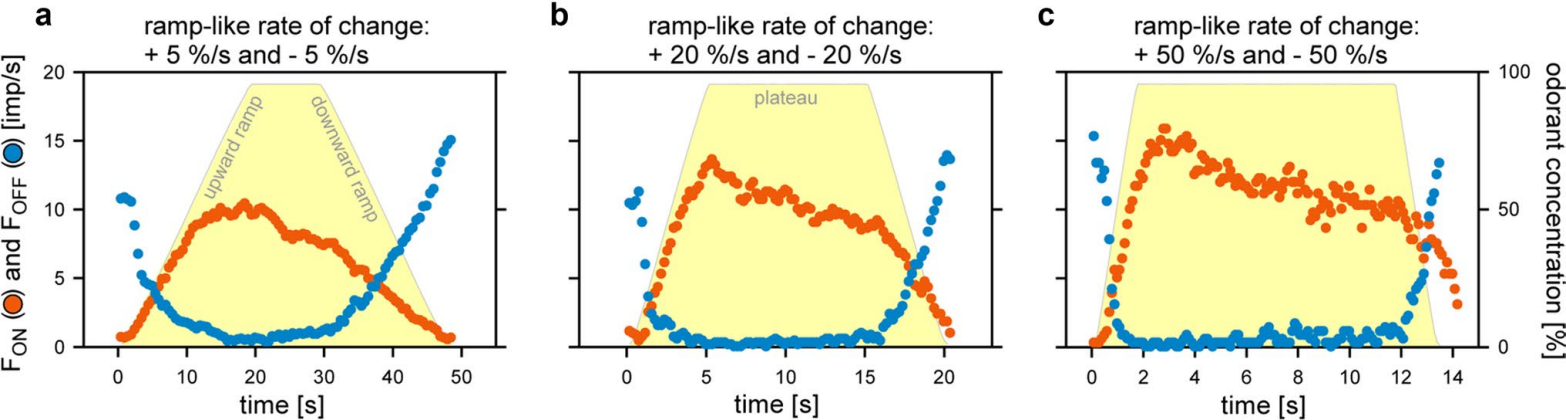
Responses of a pair of ON ORN (orange) and OFF ORN (blue) recorded simultaneously from the same sensillum during linear, ramp-like changes in the concentration of the odorant of lemon oil. **a**–**c** Time courses of ramp-like upward (positive sign) and downward (negative sign) concentration changes at three different rates (yellow areas), and the corresponding time courses of the impulse frequencies of the ON and OFF ORNs. Bin widths for impulse counts were 0.5 s for 5%/s ramps, 0.2 s for 20%/s ramps, and 0.1 s for 50%/s ramps. Adapted from Tichy et al. (2016)

The resolving power of an ORN is its ability to discriminate changes in odorant concentration. The differential sensitivity or gain of response is insufficient for this task. The differential sensitivity is given by the slopes of the regression planes that described the relation between the instantaneous concentration, its rate of change, and the response (Fig. 5). Nonetheless, slope and height of such planes poorly indicate the amount of scatter of individual responses. The resolving power, however, can be calculated from the differential sensitivity and the degree of scatter. Here, we consider a single pair of responses of a single ORN of average differential sensitivity and response scatter. How large must the difference between two concentrations be for the ORN to differentiate them with a 90% probability (Burgstaller and Tichy 2011)? The smaller the difference, the greater is the discriminatory ability. Slowly oscillating concentration changes are not suitable for determining the resolving power, because the periods during which concentration changes at constant rates are very short. We, therefore, tested ramp-like concentration changes consisting of a linear increase in concentration from clean to odorant-saturated air, followed by linear decrease to clean air. Ramp durations ranged from 2 to 20 s, generating constant upward ramps at rates between + 5 and + 50%/s, and correspondingly, constant downward ramps between − 5 and − 50%/s (Fig. 8). Impulse frequencies of the ON and OFF ORNs have been determined by counting action potentials during different intervals, ranging from 0.1 s for slow ramps of 50%/s to 0.5 s for fast ramps of 5%/s, resulting in 20–40 data points per ramp, respectively.

**Fig. 9:**
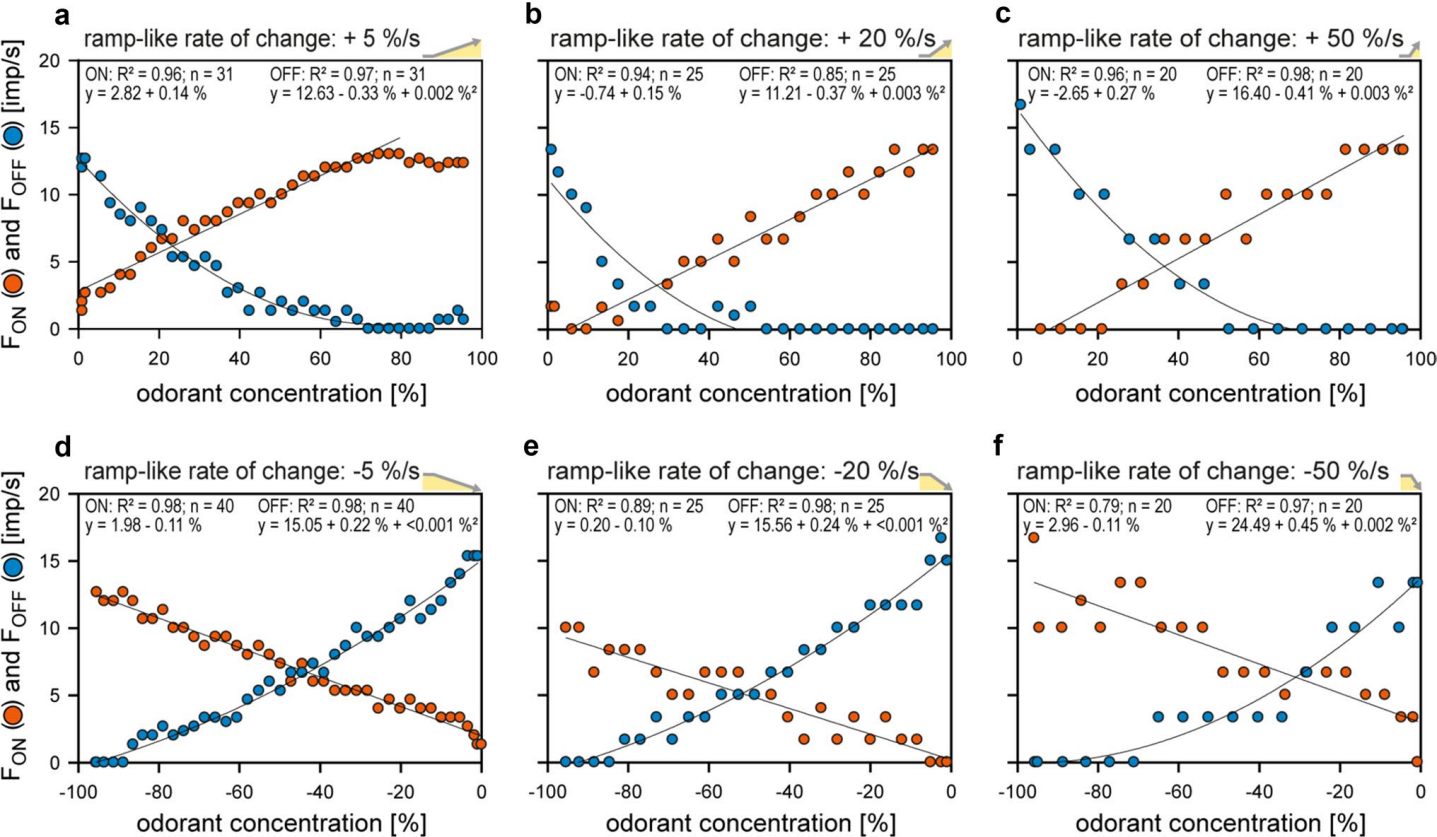
Responses of a pair of ON ORN (orange) and OFF ORN (blue) recorded simultaneously from the same sensillum during linear, ramp-like concentration changes at three different rates of change plotted as functions of instantaneous odorant concentration. Linear and parabolic regressions were used to approximate the stimulus–response relationships for the ON and OFF ORNs, respectively. The negative frequency values are due to the downward direction of the concentration change, resulting in an increase in impulse frequency of the OFF ORN. Bin widths for impulse counts were 0.5 s for 5%/s ramps, 0.2 s for 20%/s ramps, and 0.1 s for 50%/s ramps. *n*, number of points per plot; *R*^2^, coefficient of determination. Adapted from Tichy et al. (2016)

**Fig. 10:**
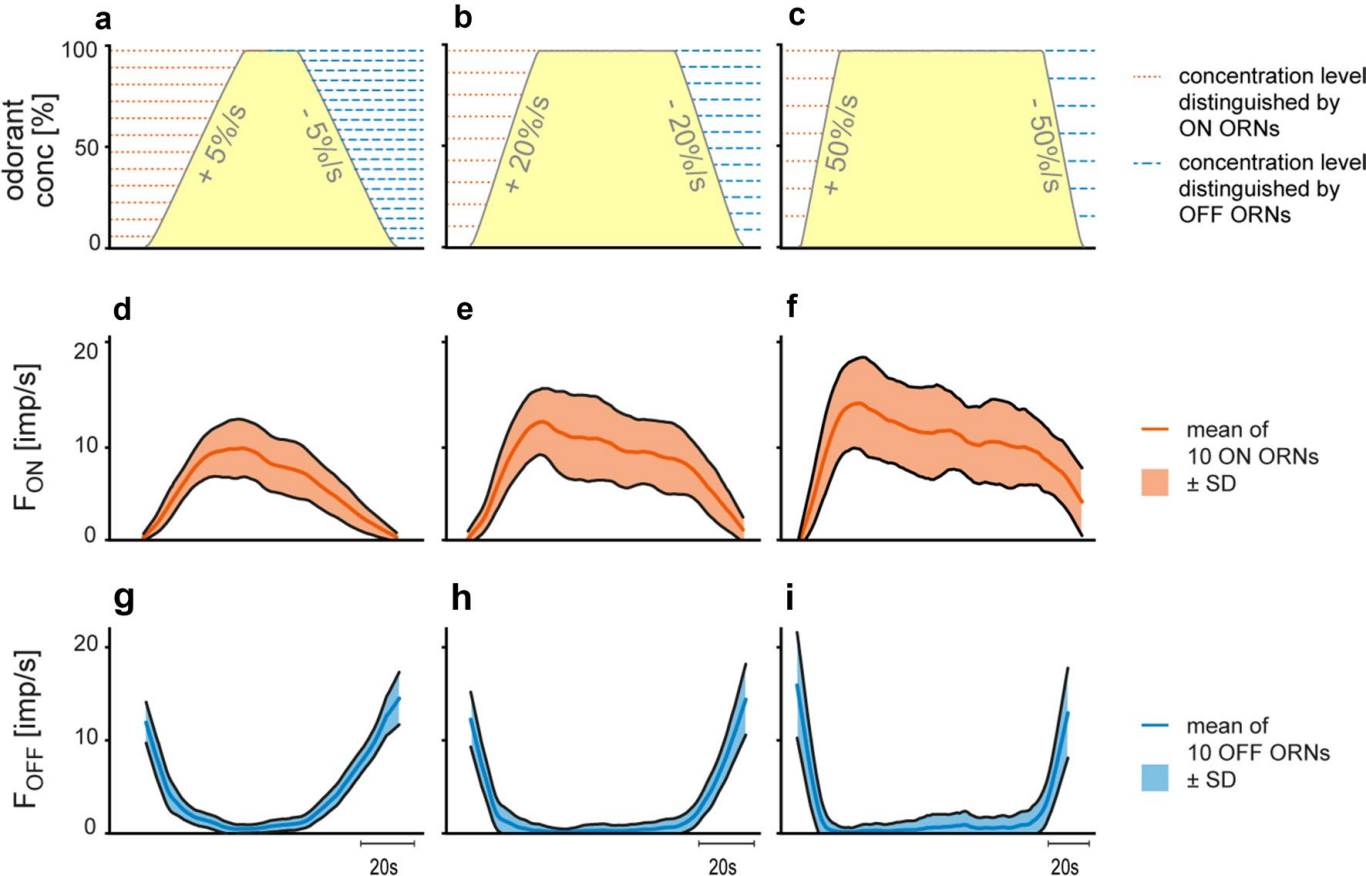
Mean responses of ten pairs of ON ORNs (orange) and OFF ORNs (blue) during linear, ramp-like concentration changes at three different rates. **a**–**c** Time courses of upward (positive sign) and downward (negative sign) concentration changes (yellow areas). Horizontal lines indicate the resolving power, i.e., the number of concentration levels that can be discriminated by the responses of the ON ORN, during a slow and continuous concentration increase, and by the responses of the OFF ORN, during a slow and continuous concentration decrease. **d**–**i** Time courses of mean frequencies. Band width indicates standard deviations of the responses values. Bin widths for impulse counts were 0.5 s for 5%/s ramps, 0.2 s for 20%/s ramps, and 0.1 s for 50%/s ramps. Adapted from Tichy et al. (2016)

In the ON ORN, upward ramps elicit a continuous increase in impulse frequency (Fig. 9a–c). The faster the rate of rise, the more rapidly the increases in impulse frequency and the higher the values achieved. Similarly, in the OFF ORN, downward ramps produce a continuous increase in impulse frequency. The faster the decline, the faster impulse frequency increased (Fig. 9a–c). The frequency curves of the ON ORNs were described by linear regressions; those of the OFF ORNs by parabolic regressions. The range of variations of the responses plays a key role for reliably encoding concentration changes. Figure 10 illustrates the time course of the mean responses with their standard deviations obtained from ten ON and ten OFF ORNs for the set of three upward and downward ramp-like concentration changes. In the ON ORN, the response variability increases with rising rates of change (Fig. 10d–f). In the OFF ORN, falling rates of change influence response variability less (Fig. 10g–i). The lower the variability, the greater the amount of information conveyed by the responses. Calculating the resolving power (Tichy et al. 2016) indicates that the ability of both ORNs to detect differences in the instantaneous concentration decreases with increasing rate of concentration change. For upward concentration ramps, the resolving power of the ON ORN is 8% for rates of + 5%/s, 11% for rates of + 20%/s, and 14% for rates of + 50%/s. The respective values of the OFF ORN are more accurate: 5, 7, and 9%. For downward concentration ramps, the resolving power of the OFF ORN is 5% for rates of − 5%/s, 8% for rates of − 20%/s, and 13% for rates of − 50%/s. The respective values of the ON ORN less precise: 11, 23, and 85%. These values enable calculating the maximum number of different concentration levels an ORN can distinguish when odorant concentration changes at constant rates from zero to saturation or vice versa. The results show that when exposed to upward ramps, the ON ORN can distinguish 12 concentration levels at a rate of + 5%/s, 9 levels at a rate of + 20%/s, and 7 levels at a rate of + 50%/s. The levels in the OFF ORN for downward ramps are: 20 at − 5%/s, 12 at – 20%/s, and 7 at − 50%/s (Fig. 10a–c).

The finding that the resolving power for instantaneous concentration values decreases at increasing rates of continuous upward and downward concentration change (Tichy et al. 2016) is confirmed by the resolving power for transient upward or downward concentration changes (Burgstaller and Tichy 2011). During concentrations steps, however, the rate of change was too fast to measure accurately. Since 0.1 s was necessary to replace the clean-air stream by the odorant-saturated air stream, this period was used to estimate transition time. Within the initial 0.1 s of the concentration step, a + 50% transient change causes an average rate of change of + 500%/s, and a 100% transient change an average rate of + 1000%/s. In the ON ORN, the resolution for a 50% rapid concentration increase—corresponding to a + 500%/s rate of change—was 28%. This value is low compared to 8% for the + 5%/s ramp or 14% for the + 50%/s ramp. In the OFF ORN, the value for a − 50% transient concentration decrease—corresponding to a − 500%/s rate of change—was 40%. This is very low compared to 6% for the − 5%/s ramp or 14% for the − 50%/s ramp.

The increased performance of both ORNs with decreasing rate of concentration makes them well suited for detecting slow and continuous concentration changes. One can assume that the ON and OFF ORNs identify and encode the onset and offset slopes of odorant pulses, respectively. Together with the pulse concentration and pulse duration, and in combination with the duration of the intervals between odorant pulses and the interval concentration, the ORNs apparently reflect the spatial–temporal odorant landscape (Moore and Atema 1988, 1991; Zimmer-Faust et al. 1995; Finelli et al. 1999). Unsurprisingly, the rate of concentration change covaries with the resolving power, because precision takes time. In behavioral choice responses, the relevant parameters are speed and accuracy, and their relationship is well known as the speed–accuracy trade-off (Klein 2001; Uchida and Mainen 2003; Heitz 2014). This trade-off is an adjustable process balancing between the correct decisions and wasted time (Heitz 2014). The balancing effect on the choice behavior has been studied in humans and in many animals, ranging from rat to bee (Heitz 2014). In mammals, odorant detection is phase-locked to the sequences of the sniffing cycle. The sampling time of just one sniff (200 ms) suffices for a rat to discriminate two pure odorants and their mixtures. Longer sampling periods or more sniffs did not improve odorant quality discrimination (Uchida and Mainen 2003). By contrast, the visual discrimination of rates is better in rats faced with natural images in slow trials and long reaction time versus fast trials and short reaction time (Reinagel 2013). The ability of the ORNs to more precisely discriminate concentration changes if the rate of change is slow corresponds with image discrimination in rates. Longer periods of data acquisition or longer stimulus durations ensure more accurate detection. Cockroaches differentiate between small concentration changes as long as the rate of change is slow. At high rates, however, the signal is detected at the expense of accurate concentration. The trade-off between speed and accuracy is a compromise between confidence and sampling time. It involves a tolerable shortcoming with desirable savings in time.

No data are available on the sampling time of cockroach ORNs. This issue was already addressed in the early electrophysiological experiments on the pheromone receptors of the moth *Bombyx mori*. High-concentration pheromone pulses elicit phasic responses that terminate within 250 ms; the interpretation was that the duration of the phasic response suffices for detecting the concentration information (Kaissling 1986). Lobsters and other marine crustaceans flick a pair of antennules, so that the chemosensory hairs located there capture odorant molecules from the surrounding water. The maximal flick rate of 5 per second reflects a 200 ms odorant sample. This period corresponds to the duration of onset slopes of odorant pulses, creating the concentration pattern of the odorant landscape (Atema 1995, 1996).

The steepness of the pulse onset slopes has been described in detail only for aquatic odorant plumes (Wolf et al. 2004). Their dispersal was measured using fluorescent dyes or tracers at high temporal resolution. Diagrams of the time course of the odorant concentration are available and provide information on the spatial distribution of pulse slopes, pulse concentrations, and pulse durations. Unfortunately, direct measurements of the rate of concentration change are lacking. Nonetheless, the rate of change can be estimated with eye and ruler from the published diagrams. Accordingly, the onset slopes cover concentration rates between 4%/s (25 µM/s dopamine) and 100%/s (400 µM/s dopamine) (Fig. 4, in Atema 1996).

Apart from the cockroach’s ORNs, the resolving power has not yet been examined in arthropods. The ability to discriminate slow and continuous concentration changes better than rapid pulse-like changes probably reflects the demands that food-odorant signals places on the animal. Thus, cockroaches show one possible solution to the problems posed by a given olfactory environment in terms of physiological, morphological, and behavioral systems. This includes the need for parallel processing in the olfactory system which is immediately appreciated when one considers the different parameters that are contained in olfactory signals and the multitasking abilities of the brain in which it handles many of these simultaneously. The next section addresses the strategy of processing in parallel systems.

## The separation of encoding temporal information of the odorant signal begins directly in olfactory receptor neurons (ORNs)

The processing of visual information in the CNS is widely held to be a progression from the general to the particular: from a peripheral code in which the level of excitation of retinal neurons represents raw data on the quality and intensity of the visual stimulus, to a cortical code in which individual neurons signal the presence of bars, edges, or movement in a certain direction. Observations made by Barlow (1961, 1969) lead to the conclusion that the sense organs bring a vast amount of information to the gates of the CNS, out of which the central synapses select what they need and discard the redundant details. This idea is supported by the vastly great number of the first-order versus second-order sensory neurons; the latter further outnumbering third-order neurons. The inherent limit of serial processing is that a central stage of response selection can process only one task at a time. Models of behavior control, however, propose that two or more response selections can be processed at once and proceed in parallel. Processing sensory information via multiple parallel pathways breaks the complex representation of the external environment into distinct information streams. These parallel pathways encode simultaneously a subset of stimulus features and contribute to the specific aspects of a unified sensory percept.

In the insect olfactory system, parallel processing has been best studied in the Hymenoptera (Galizia and Rössler 2010) but without revealing simple general rules. In the honeybee, for example, a dual pathways is formed by two independent sets of glomeruli and PNs, which pass through the antennal lobe via two anatomically distinct fiber tracts, the lateral and the medial antennal lobe tracts (l- and m-ALT), to reach the mushroom bodies and the lateral horn (Abel et al. 2001; Müller et al. 2002; Kirschner et al. 2006; Galizia and Rössler 2010; Schmuker et al. 2011; Nawrot 2012; Brill et al. 2013; Rössler and Brill 2013; Carcaud et al. 2015). The main function of this dichotomy is not differentiation of odorant spectra but separate extraction and processing of different features of the odorant signal. The l-ALT PNs are characterized by fast responses to a broad spectrum of odorants, providing information about the timing or temporal structure of an odorant. The m-ALT PNs produce slower responses to specific odorants, encoding information about odorant identity. Similar to the non-spatial “what-component” and the spatial “where-component” processing pathways in the vertebrate visual system (Mishkin et al. 1983; Merigan and Maunsell 1993; Milner and Goodale 2008), the two parallel m- and l-ACT pathways in the honeybee olfactory system provide “what” (quality) and “when” (temporal) information of the odorant signal, respectively (Brill et al. 2013). The role of lateral inhibition and gain control in generating the different coding properties in the m- and l-ALT was studied by implementing a computational network model to the neural circuits of the antennal lobe (Schmuker et al. 2011). This analysis suggested that the l-ALT detects and identifies odorants at low concentration, providing no concentration sensitivity, whereas the m-ALT accurately detects and tracks concentration gradients but is less engaged in odorant discrimination. This segregation trades off odorant identification for concentration discrimination.

In the cockroach’s olfactory system, two separate, parallel PN pathways have been described (Watanabe et al. 2017). The PNs originate in different areas of the antennal lobe within two distinct groups of glomeruli: the antero-dorsal (AD) and the postero-ventral (PV) group. While the AD glomeruli are innervated by the ORNs of the basiconic *swB* sensilla and connected to type 1 PNs, the PV glomeruli receive the axons of the ON and OFF ORNs located in the trichoid *swC* sensilla and transmit information via type 2 PNs to higher brain centers. Future work should evaluate whether and to what extent the temporal dynamics of the odorant stimuli contained in the responses of the ON and OFF ORNs are represented in the discharge of the type 2 PNs.

Our studies of the cockroach’s peripheral olfactory system revealed that the segregation into two pathways originates in two anatomically distinguishable sensillum types: the basiconic *swB* sensilla and the trichoid *swC* sensilla. Both types are involved in encoding the odorant of lemon oil. They share the same wall structures, which is consistent with the concept that the sensillum wall is involved in transferring particular classes of odorant molecules from the outside air to the dendritic membranes (Boeckh and Ernst 1987; Watanabe et al. 2012). The physiological significance of the variations in the sensillum’s morphology (e.g., length, diameter and surface area) is less clear. The larger trichoid *swC* sensilla compared to the smaller basiconic *swB* sensilla probably provide a larger surface area and thus more pore tubules in the wall. This would enhance the access of odorant molecules to the dendritic membrane. Larger external dimensions also suggest larger internal structures including the length, diameter, and surface area of the distal dendritic segments. The result is larger membrane areas and higher numbers of ion channels. More pore tubules in the cuticular wall and more ion channels in the dendritic membranes would improve ORN sensitivity. One expectation is that the ORNs in the trichoid *swC* sensilla are more sensitive and respond more strongly to the key odorant stimulus than ORNs in the basiconic *swB* sensilla. Unfortunately, studies of the latter sensilla type are purely qualitative.

Two separate pathways originating in the basiconic *swB* and the trichoid *swC* sensilla may have evolved, because identifying an odorant and using it for action require different operating procedures of the olfactory signals. Successfully orienting within an odorant plume requires the nervous system to calculate both instantaneous concentration and the direction and rate of concentration change. The timing of these calculations is critical. As the insect approaches the source, they do not provide a static relationship. It calls for calculating the temporal properties of the odorant signal in an insect-centered framework at the very moment that the movements are to be performed. Odorant identity must be processed quite differently. This does not require calculating the fluctuations in concentration. In fact, such calculations would be counter-productive. It would be better to recognize a complex odorant mixture regardless of the fluctuations in its components or the relationship of the mixture to other, even stronger, odorants present in the environment. A scene-based framework must serve as a reference over a lengthier time scale to allow the insect to recognize the odorants from one occasion to the next. This involves combining the current input with stored information. We propose that the information stream originating at the basiconic *swB* sensilla provides characteristics about odorant identity (“olfaction-for-identification”), whereas the trichoid *swC* sensilla stream is optimized to signal the timing of an odorant, the rate of increase and decrease in concentration, and the instantaneous value. This can be understood as a specialization for mediating the olfactory control of orientation to an odorant source (“olfaction-for-action”).

An olfactory pathway mediating “action” has been described for PNs of the macroglomerular complex (MGC) of the male hawkmoth (Lei et al. 2009). These PNs produce spontaneous activity patterns of brief bursts of impulses separated by silent periods. The bursting patterns are reversibly transformed to tonic discharging by injection of a GABA_a_-receptor antagonist, bicuculline, into the MGC. Bicuculline treatment has no effect on the simultaneously recorded activities of sex pheromone ORNs. It seems that the drug does not disrupt the ability of MGC-PNs to receive the input pheromone signal, but it modifies their temporal response patterns. When navigating in a turbulent odorant plume, these MGC-PNs are suited for resolving and enhancing the intermittent sex pheromone stimulation. Wind tunnel experiments demonstrated that, after injecting this drug into the AL, male moths cannot locate the source of the sex pheromone. This modification in the orientation behavior was explained by the disruption of the patterned activity of MGC-PNs caused by bicuculline. The study revealed a link between the bursting activity patterns of the MGC-PNs and pheromone orientation behavior of the male hawkmoth. The authors conclude that these MGC-PNs provide a potential neural substrate essential for odorant plume tracking rather than for odorant identification.

Encoding and processing olfactory information in the cockroach appear to differ considerably from the honeybee. In the latter, the outflow from the AL is segregated into two tracts, which help to process different aspects of the odorant signal. Concerning their functional roles, the l-ACT may correspond with the basiconic *swB* sensilla stream-mediating odorant identification, and the m-ACT with the trichoid *swC* sensilla stream encoding the rate of concentration change. Different coding and processing strategies may exist in odorant-guided orientation, which have evolved in different contexts and under different conditions. A foraging honeybee, flying from flower to flower, is confronted with different tasks than a cockroach running on the floor of a warehouse or on the ground outdoors, searching for garbage or windfall. Nevertheless, both insects have to solve the tricky task of encoding and simultaneously processing information about the identity of the odorant signal and its temporal properties. Although our studies have been focusing the division of odorant coding between the basiconic *swB* and the trichoid *swC* sensilla streams, it is clear that the two streams must interact closely in everyday life. Indeed, the ability to identify odorants would never have evolved unless it had some adaptive behavioral value. Future studies that systematically examine this issue at higher levels of the olfactory pathways are needed to establish these possibilities as facts. That research must determine how much information about the temporal properties of food-odorant stimuli can be extracted from the discharge rates in the pathways which they activate.

As described above, the ratio between the sensilla involved in processing “olfaction-for-action” and “olfaction-for-identification” is 1:4.5. The presence of a low percentage of ON and OFF ORNs does not necessarily denote a minor function for odorant coding. One would expect that the larger the number of ORNs is, the better the performance. However, the accuracy may also improve with sharper tuning. Theoretical analysis has shown that the relationship between tuning width and accuracy depends crucially on the range of stimulus variables to be encoded by a particular neuron (Zhang and Sejnowski 1999). This strategy, referred to as ‘sparse coding’, offers some advantages: it explicitly reveals the parameters encoded in the neural activity and forwards complex stimuli in an easily understandable and extractable form to higher levels of processing (Olshausen and Field 2004). To encode efficiently, a neural system must be able to change its coding strategy as the distribution of the characteristic stimulus features changes. A large number of ORNs may help to accomplish such tasks. Conversely, an exact shape of the tuning function may provide the best performance of processing the steepness of odorant pulses with small numbers of ORNs. In addition to coding efficiency, narrow sensory tuning is also very much depending on the precision required of the downstream motor response that contributes to generating actions (Salinas 2006). The amount of reliable information fed into the brain is involved in the question of optimal sensory tuning and their answers would indicate the ORN’s role during odorant tracking.

## The contribution of the ON and OFF olfactory receptor neurons (ORNs) to orientation

Signals arising in the natural world are varying continuously in time—sound pressure at the eardrum, light intensity in a region of the visual field, and odorant concentration at the insect antennae. The detection of changes in odorant concentration is a fundamental function of the olfactory sense, so that the research on the olfactory system has been devoted to the investigation of the physiological basis of encoding concentration changes. One finding in our experiments was a gain control mechanism in the ON and OFF ORNs that adjusts their operation range to match the prevailing fluctuations in odorant concentration. Unfortunately, no studies have been performed so far on the temporal concentration pattern of odorants emitted continuously by ripe citrus fruits. In indoor environments such as fruit storages, kitchens, or basement rooms, where winds do not shift frequently from one direction to another, the fruit odorant plume will be rather homogeneous and not heavily intermittent. In open areas, like forest soils or landfill sites, where turbulent air disperses the food odorant from the source, the plume will be disrupted into odorant pulses and diluted. With increasing distance from the source, the pulse slopes will become less steep and their heights will fall more slowly.

A cockroach entering a turbulent plume will be faced with frequent concentration changes. When the odorant signal is smoothly fluctuating with long pulse durations, an increased gain of the ON and OFF ORNs for the rate of change will ameliorate their ability to detect slow upward and downward concentration changes. Due to the double dependence of the responses on the instantaneous concentration and its rate of change, the cockroach will extract information on the concentration level around which the fluctuation occurs and find the areas of higher concentration with faster concentration increase. This moves the insect closer to the odorant source. Near the source, when the fluctuating periods are brief, the ON and OFF ORNs will increase the gain for the instantaneous concentration. The final approach requires detecting small differences in concentration rather than small differences in the rate of change. Gain control of both ORNs balances between sensitivity to instantaneous concentration and sensitivity to the rate of change. The literature provides only very few examples of pulse slope detectors. This may reflect the difficulty in controlling and measuring changes in odorant concentration at different rates and should not be construed as a lack of their importance.

## Abbreviations

AD: Antero-dorsal group of glomeruli
AL: Antennal lobe
ALT: Antennal lobe tract
dw: Double-walled sensillum
MGC: Macroglomerular complex
ORN: Olfactory receptor neuron
PN: Projection neuron
PV: Postero-ventral group of glomeruli
sw: Single-walled sensillum
